# Enhanced Electrochemiluminescence from Ruthenium-Tagged Immune Complex at Flexible Chains for Sensitive Analysis of Glutamate Decarboxylase Antibody

**DOI:** 10.3390/bios15010047

**Published:** 2025-01-15

**Authors:** Yuyao Zhang, Li Qian, Qian Zhang, Yu Li, Yu Liu, Dechen Jiang

**Affiliations:** 1 State Key Laboratory of Analytical Chemistry for Life Science, School of Chemistry and Chemical Engineering, Nanjing University, Nanjing 210092, China; 502022240162@smail.nju.edu.cn (Y.Z.); 502023240144@smail.nju.edu.cn (Q.Z.); dechenjiang@nju.edu.cn (D.J.); 2 Sir Run Run Hospital, Nanjing Medical University, Nanjing 211100, China; drqianli@njmu.edu.cn; 3 School of Materials Engineering, Jingling Institute of Technology, Nanjing 211169, China

**Keywords:** electrochemiluminescence, flexible PEG chain, GADA, ruthenium complex, immunosensors

## Abstract

Herein, a sensitive electrochemiluminescence (ECL) immunosensor is designed by immobilizing ruthenium-tagged immune complexes at flexible poly-ethylene-glycol (PEG) chains on the electrode surface, which offers more freedom for the collision of the ruthenium complex at the electrode during the initial ECL reaction. The electrochemical characterizations confirm the loose structure of the assembled layer with the immune complex, providing an increase in the current and the resultant enhanced ECL emissions. Comparing the sensors with the rigid structure, a 34-fold increase in the maximal ECL emission is recorded when PEG3400 is used as a linker. Using the optimized protocol, the prepared immunosensor exhibits a wide-ranging linear response to the model antibody (glutamate decarboxylase antibody) ranging from 10 pg/mL to 10 ng/mL. The detection limit is almost two orders lower than the value using the classic enzyme-linked immunosorbent assay, which offers a new design to enhance ECL emissions and the resultant analytical performance.

## 1. Introduction

Type 1 diabetes mellitus (T1DM) is a chronic autoimmune disease caused by the combination of susceptibility genes and environmental factors, resulting in damage to pancreatic β cells [1,2,3]. In recent years, there has been a global increase in the prevalence and incidence of type 1 diabetes, which is commonly diagnosed during early adulthood and adolescence [4,5,6,7,8]. Currently, the classic markers for the diagnosis of T1DM include the glutamate decarboxylase antibody (GADA), protein tyrosine phosphatase antibody (IA-2A), insulin antibody (IAA), islet cell antibody (ICA) and transporter 8 antibody (ZnT8A) [9,10]. Among these antibodies, GADA exhibits the highest sensitivity and specificity and, thus, serves as an important immune index for T1DM diagnosis [11,12,13]. The role of GADA is involved in the pathogenesis of immune-mediated (type 1) diabetes. Immune-mediated diabetes constitutes 5–10% of patients with diabetes, and GADA was identified in 92% of adult autoimmune diabetic patients. Patients with GADA-positive diabetes have a higher risk of cognitive decline compared to those with GADA-negative type 2 diabetes of similar diabetic severity. GADA might mediate cognitive dysfunction by disrupting γ-aminobutyric acid (GABA) production and might contribute to dementia in diabetics [14]. The radioligand binding assay (RBA), owing to superior detection sensitivity, has been utilized as an internationally recognized standardized method for GADA detection [15,16]. However, due to its high cost, potential radioactive contamination, and complex and time-consuming procedures, this technology faces a challenge in future applications. Therefore, the need to develop a sensitive and environmentally friendly assay for the rapid and efficient analysis of GADA is still urgent.

The electrochemiluminescence (ECL) technique is the combination of electrochemical reactions and chemiluminescence, wherein the electrochemical reaction generates active intermediates and excited luminous species [17,18,19]. Subsequently, the excited luminous species returns to its ground state and emits luminescence. Due to the absence of a light source throughout the entire process, the ECL method overcomes the background signal interference and offers the advantage of high sensitivity and a rapid response time [20,21]. Consequently, ECL-based assays have been widely applied in biosensing and clinical tests [22,23]. Tris(2,2′-bipyridine)ruthenium(II) (Ru(bpy)_3_^2+^) as the luminophores and tri-*n*-propylamine (TPrA) as the co-reactant reagent are popular in the ECL-based clinical immunoassay [22,24,25]. Classically, the ECL immunosensor captures the target antigen by a specific antibody immobilized on the electrode, and then the Ru(bpy)_3_^2+^ labeling antibody targets the antigen to assemble a “sandwich” structure. A few mechanisms have been established to explain the generation of ECL by Ru(bpy)_3_^2+^/TPrA [26]. Considering the immobilized Ru(bpy)_3_^2+^ at the immune complex a certain distance away from the electrode surface, the direct electron transfer at Ru(bpy)_3_^2+^ is not favored. In most cases, only TPrA is oxidized to the radical cation TPrA^+•^ on the electrode surface (Equation (1)) and is deprotonated to form TPrA^•^ radical (Equation (2)). This radical can reduce the luminophore Ru(bpy)_3_^2+^ to form Ru(bpy)^3+^ (Equation (3)). Subsequently, Ru(bpy)^3+^ is oxidized by TPrA^+•^ to generate Ru(bpy)_3_^2+*^ (Equation (4)), which emits light (Equation (5)). The reactions of this “visiting pathway” are expressed as follows:

(1) $$TPrA\;\to {TPrA}^{+•}+e^{-}$$

(2) $${TPrA}^{+•}\;\to \;{TPrA}^{•}+H^{+}$$

(3) $${Ru(bpy)}_{3}^{2+}+{TPrA}^{•}\;\to \;{Ru(bpy)}_{3}^{+}+products$$

(4) $${Ru(bpy)}_{3}^{+}+{TPrA}^{+•}\;\to \;{Ru(bpy)}_{3}^{2+*}+TPrA$$

(5) $${Ru(bpy)}_{3}^{2+*}\;\to \;{Ru(bpy)}_{3}^{2+}\;\to hv$$

Despite significant achievements in the development of ECL-based immunosensors, further enhancement of ECL emissions and the improvement of sensor performance are still needed for the better analysis of low-abundant proteins in the serum. A variety of methods have been explored to enhance ECL emissions [27]. For example, developing high-performance ECL emitters could improve electron transfer efficiency or reduce non-radiative transitions during the process of emission to enhance ECL performance [28,29,30]. Through the “confinement effect”, confining the emitters and co-reactants to a limited spatial field can significantly improve ECL efficiency by increasing the local concentration of the reactants [31,32]. The introduction of suitable conductive materials enhances the interface conductivity and promotes electron transfer, thus amplifying the ECL signal [33]. In addition, ECL signals can also be amplified by ECL-resonance energy transfer technology and surface plasma-enhanced ECL methods [34,35]. However, these strategies only focus on increasing the electron transfer rate and have not yet improved ECL by the realization of more collisions between the Ru(bpy)_3_^2+^ and electrode surfaces.

**Figure 1:**
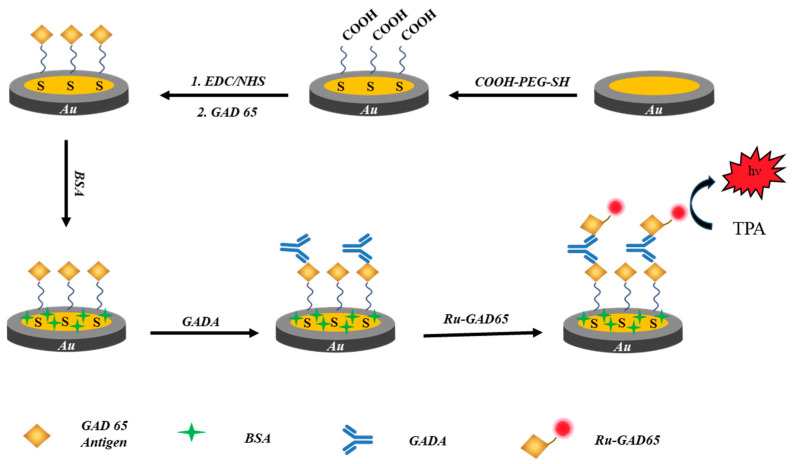
Schematic illustration of the construction process of the ECL immunosensor with PEG chains.

In this work, a sensitive GADA ECL immunosensor was designed by immobilizing ruthenium-tagged immune complexes at flexible poly-ethylene-glycol (PEG) chains, which is proposed to induce the greater freedom of the ruthenium complex for the collision at the electrode, and thus, enhance ECL detection. As illustrated in Figure 1, PEG with a long-chain structure is self-assembled at the gold electrode, which allows the linker to immobilize the glutamic acid decarboxylase 65-kilodalton isoform (GAD65) as well as the recognition layer. GADA as the target analyte is introduced by exploiting specific binding interactions between GAD65 antigens and antibodies to form a sandwich complex. Then, Ru(bpy)_3_^2+^ as the luminophore is labeled at GAD65 to form an immune complex, which emits luminescence as the signal. Differing from the previously reported immune layer with the rigid structure, the established structure with a long-chain PEG linker can lead to the collapse of the sandwich structure so that the ruthenium complex has a greater possibility of colliding with the electrode surface. The addition of direct electron transfers between Ru(bpy)^2+/3+^ in the ECL reaction could enhance ECL emissions, and thus, the sensitivity of detection to GADA could be improved. The achievement of this novel sensor design will offer a new strategy for the fabrication of sensitive immunosensors that benefit clinic tests in the future.

## 2. Materials and Methods

### 2.1. Reagents and Apparatus

Gold electrodes were provided by Eaglenos Co., Ltd. (Nanjing, China). Thioglycolic acid (TGA) was purchased from Sinopod Chemical Reagent Co., Ltd. (Shanghai, China). The GAD65 antigen and bio-GAD65 antigen were obtained from Genscript Bio Co., Ltd. (Nanjing, China). GADA, IA-2A, IAA, and ZnT8A were purchased from Bioss Biotechnology Co., Ltd. (Beijing, China). All the other chemicals were purchased from Sigma-Aldrich Chemical Co. (St. Louis, MO, USA). The chemicals were of analytical reagent grade and used as received. All aqueous solutions were prepared using ultrapure water (Milli-Q, Millipore, Billerica, MA, USA).

ECL detection was measured by an electrochemical luminescence detector (MPI-A, Xi’an Remex Analysis Instruments Co., Ltd., Xi’an, China). The electrochemical measurements were conducted by electrochemical workstations (CHI 750e, Chenhua Instrument Co, Ltd., Shanghai, China). The surface morphology of the electrodes was acquired using field emission scanning electron microscopy (JSM-7800F, JEOL, Tokyo, Japan).

### 2.2. Fabrication of the Immunosensor

The Au electrodes were cleaned three times using an ultrasonic method in ethanol and an aqueous solution. To prepare the carboxylated surface, a volume of 10 μL of HS-PEG(n)-COOH (10 μM, diluted in ultrapure water) was dropped onto the Au electrode and reacted at 4 °C overnight. Then, the electrodes were washed using demineralized water to remove excess HS-PEG(n)-COOH. EDC (*N*-ethyl-N′-(3-(dimethylamino)propyl)carbodiimide) and NHS (*N*-hydroxysuccinimide) were dissolved in an MES (2-(*N*-morpholino)ethane sulfonic acid) buffer to obtain 0.1 M solutions. Afterward, a volume of 10 μL of a mixture of EDC/NHS (1:1) solution was added at the Au electrode for the activation at 4 °C for 30 min. After that, the electrode was rinsed with 0.1 M phosphate-buffer saline (PBS) containing 0.05% Tween 20. Finally, 5 μL of the 10 μg/mL GAD65 antigen (Ag) was added to the Au electrode with incubation at 4 °C for 2 h. To minimize non-specific adsorption, the Au/HS-PEG(n)-COOH/Ag bioconjugate was blocked by incubation in 5 μL 3% BSA solution at 37 °C for 30 min. Lastly, the modified electrode was washed with 0.1 M PBS (pH 7.4) to remove excess BSA and stored at 4 °C in wet conditions for further use.

The Ru(bpy)_3_^2+^-labeled antigens were prepared as follows: 1 mg/mL Ru(bpy)_3_^2+^ and 0.1 mg/mL streptavidin were reacted with stirring at 4 °C for 2 h. The mixed solution was purified by ultrafiltration using a 10 K molecular weight cutoff membrane. The concentrated streptavidin-modified Ru(bpy)_3_^2+^ complex was diluted to 20 μg/mL with PBS (pH 7.4) and incubated with the biotinylated GAD65 antigen at 37 °C for 30 min to obtain the product of the Ru(bpy)_3_^2+^-labeled GAD65 antigen (Ru-Ag). For the prepared immune complex with the sandwich structure, 5 μL of the sample solution involving different concentrations of GADA was dropped onto the electrodes and incubated at 37 °C for 1 h to obtain Au/HS-PEG(n)-COOH/Ag/GADA. Then, 5 μL of the diluted Ru-Ag solution was dropped on the electrode surface at 37 °C in the dark for 1 h. The excess Ru-Ag was removed by the washing solution. Ultimately, the sandwich immunosensor (Au/HS-PEG(n)-COOH/Ag/GADA/Ru-Ag) was stored in wet conditions at 4 °C for further ECL detection. The sensors can only be used for one experiment and could not be rehydrated before the other test.

### 2.3. Electrochemical Characterization of the Immunosensor

Cyclic voltammetry (CV) and electrochemical impedance spectroscopy (EIS) measurements of the immunosensors were obtained using the electrochemical workstation. The Au electrode, Ag/AgCl wire, and Pt wire were used as the working, reference, and counter electrodes, respectively. CV measurements were taken between −0.2 and 0.8 V with a scan rate of 50 mV/s. EIS measurements were taken in a frequency range of 100 KHz to 0.01 Hz. The electrolyte solution used for the EIS measurements was 5 mM K_3_[Fe(CN)_6_]/K_4_[Fe(CN)_6_] (1:1) in PBS (pH 7.4). To ensure operation within the linear current−voltage range, the AC (potential amplitude) was adjusted to ±5 mV relative to the open circuit potential. Throughout all measurements, the initial open circuit potential remained stable at 399 ± 1 mV, with minimal fluctuations of less than 5 mV observed during long-term faradaic EIS measurements. The EIS measurements were conducted at room temperature.

### 2.4. ECL Measurements

The prepared electrode was placed in phosphate buffer (PB, pH 7.4) containing 0.1 M tri-*n*-propylamine (TPrA) as the co-reactants. The ECL responses were measured with a photomultiplier tube (PMT). The voltage of the PMT was applied at 700 V. The scan potential was set from 0 to 1.5 V with a scan rate of 500 mV/s. For the detection of GADA in serum, the sample source and the ethics were provided by the Sir Run Run Hospital of Nanjing Medical University (2022-SR-S038), and the experimental process fully complied with ethical approval. Serum samples were diluted 100 times with phosphate buffer (7.4), and the concentration of GADA in serum was measured by the ECL method with the standard addition method.

## 3. Results

### 3.1. Characterization of the Immune Complex at Flexible Chains

**Figure 2:**
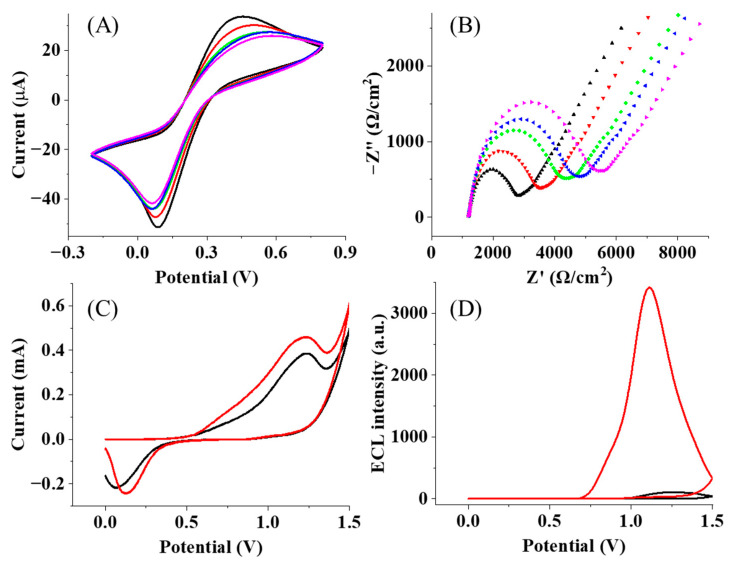
(**A**) CVs of the immunosensor fabrication in 0.1 M PBS (pH = 7.4) with the potential range from −0.2 to 0.8 V and (**B**) EIS characterization of the immunosensor fabrication in 5.0 mM K_3_Fe(CN)_6_/K_4_Fe(CN)_6_ at the frequency range from 0.01 Hz to 10 KHz. Blank line: Au/HS-PEG(3400)-COOH; red line: Au/HS-PEG(3400)-COOH/GAD65 antigen; green line: Au/HS-PEG(3400)-COOH/GAD65 antigen/BSA; blue line: Au/HS-PEG(3400)-COOH/GAD65 antigen/BSA/GADA; purple line: Au/HS-PEG(3400)-COOH/GAD65 antigen/BSA/GADA/Ru-GAD65. (**C**) CVs and (**D**) ECL intensity of the immunosensor with TGA (blank line) and HS-PEG(3400)-COOH (red line) as the connection chains with 100 mM TPrA in 0.1 M PBS.

Following the classic self-assembled strategy to prepare the immunosensor, PEGs with different lengths function with the thiol group that reacts with the Au surface for the initial assembling. Then, the surface is exposed to EDC/NHS for the activation of the carboxylic acid group. The formed amine-reactive intermediate, such as a succinimidyl ester group, can further react with the amino group and GAD65 antigen, which is bound with the corresponding GADA and Ru-Ag to form the immune complex. Then, the BSA acts as a blocking agent, which can prevent the binding of antibodies and non-specific proteins in order to avoid non-specific interactions and minimize false positive results. The whole assembled process is illustrated in Figure 1. To monitor the assembling process, electrochemical probes, [Fe(CN)_6_]^3−/4−^, were employed to display the electrochemical behaviors of the Au electrode with different layers. The serial addition of PEG, GAD65 antigen, GADA, and Ru-Ag continually introduced the insulting layers at the Au electrode, resulting in more hindrance for the penetration of [Fe(CN)_6_]^3−/4−^ to the electrode surface. When the antigen and antibody were successively immobilized onto the Au electrode, the redox peak currents decreased in succession, as shown in Figure 2A, because the electron transfer rate was hindered by the poorly conductive proteins. The EIS characterization in Figure 2B further exhibits the gradual increase in the semicircle in the spectroscopies, supporting more insulating layers at the electrode and the resultant elevation of the impedances.

After the formation of the immunosensor at the Au electrode, TPrA as the co-reactant was added into the solution to react with the ruthenium complex, generating ECL emissions. To clearly demonstrate the role of the PEG chain in the ECL process, TGA with a short chain and rigid structure was chosen to fabricate another immunosensor. To keep the same amount of Ru(bpy)^2+^ at the electrode, the same concentrations of PEG and TGA should be used. Compared with the CV trace from the TGA immunosensor, the PEG immunosensor demonstrated an increase in the peak current at 1.2 V, as shown in Figure 2C. It is important to note that the rigid structure of TGA makes it difficult for Ru(bpy)_3_^2+^ to directly contact the electrode. As a result, only TPrA in a solution can be directly oxidized at the electrode surface, and ECL is generated through the visiting pathway [36]. In this case, the oxidation current (Figure 2C, black line) primarily originates from the oxidation of TPrA and the gold electrode surface, while the reduction in current is mainly contributed to by the reduction in oxidized species on the gold electrode surface [37]. However, the long-chain PEG increases the diffusion distance of the TPrA radical from the electrode surface to the ruthenium complex, and thus, less current should be observed if the “visiting pathway” is the only route. Accordingly, the direct electron transfers between Ru(bpy)_3_^2+/3+^ must occur, which are ascribed to the flexible PEG chain. In the presence of this long and flexible PEG chain, the immune complexes are not vertically positioned at the electrode surface. Some collapsing of the immune layer will occur, leading to direct contact with the ruthenium complex at the electrode. Consequently, the loss of electrons at TPrA, Ru(bpy)_3_^2+^, and the gold surface starts to simultaneously contribute to a greater oxidation current (Figure 2C, red line). In this case, in addition to the visiting pathway, Ru(bpy)_3_^2+^ species that directly contact the electrode surface generate ECL through the oxidative-reduction route [36], the reactions of which are given as follows:

(6) $$TPrA-e^{-}⟶\;{TPrA}^{+•}\;⟶\;{TPrA}^{•}+H^{+}$$

(7) $$Ru{\left(bpy\right)}_{3}^{2+}-e^{-}\;⟶Ru{\left(bpy\right)}_{3}^{3+}$$

(8) $$Ru{\left(bpy\right)}_{3}^{3+}+{TPrA}^{•}\;⟶\;Ru{\left(bpy\right)}_{3}^{2+•}+products$$

(9) $$Ru{\left(bpy\right)}_{3}^{2+•}\;⟶\;Ru{\left(bpy\right)}_{3}^{2+}+h\nu$$

Meanwhile, the ECL emissions from PEG and TGA immunosensors are recorded and shown in Figure 2D. The 34-fold increase in the ECL intensity is observed and confirms the accelerated ECL process. More importantly, the ECL enhancement observed at the PEG immunosensor facilitates the measurement and might improve the detection sensitivity.

### 3.2. Optimization of the Fabrication Process

**Figure 3:**
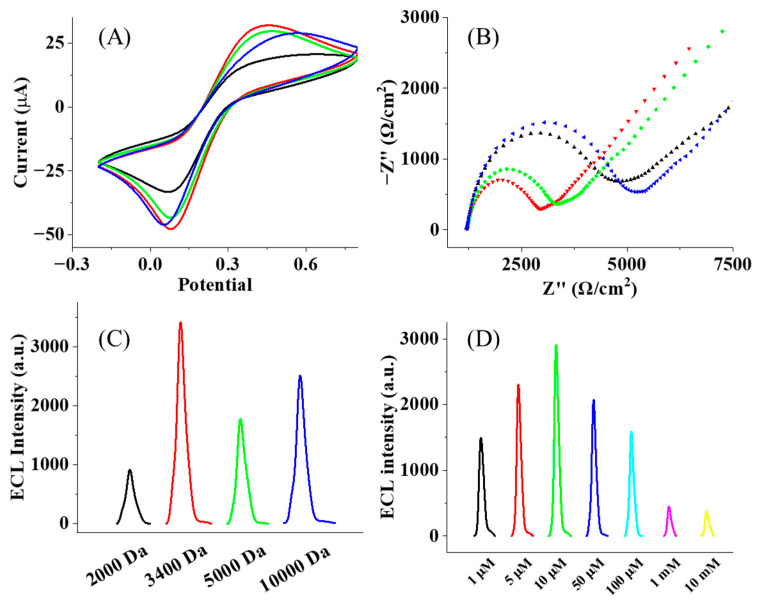
(**A**) CVs of immunosensors with PEG length and (**B**) EISs of immunosensor with PEG length (black line: 2000 Da; red line: 3400 Da, green line: 5000 Da and blue line: 10,000 Da). (**C**) ECL responses of immunosensor with PEG length (Mw ~ 2000, 3400, 5000 and 10,000 Da); (**D**) ECL intensity of immunosensors with different concentrations of SH-PEG(3400)-COOH (black: 1 μM; red: 5 μM; green: 10 μM; blue: 50 μM; cyan: 100 μM; magenta: 1 mM; yellow: 10 mM).

The length of PEG will affect the collision possibility of the ruthenium complex at the electrode, which should be optimized to achieve the best condition for the ECL reaction. Accordingly, PEG with different molecular weights (M.W. 2000, 3400, 5000, and 10,000 Da) and chain lengths are used to fabricate the immunosensors. In principle, short PEG chains tend to form a loosely packed layer on the electrode surface, which restricts electron transfer and is not favorable for the ECL process. When the chain length increases, PEG chains tend to be loose on the electrode surface, thereby facilitating an enhanced rate of electron transfer [38,39,40,41]. As shown in Figure 3A,B, an increase in the current and a decrease in the impedance are observed when the molecule weight increases from 2000 to 3400 Da. A longer PEG chain leads to the formation of a thick layer on the electrode surface, resulting in an increased tunneling distance for electron transfer and a reduced rate of electron transfer. Consequently, when the molecular weight of PEG increases from 3400 to 5000 and 10,000 Da, the current decreases are associated with an increase in the impedance. Consistent with the change in current and impedance at the electrode, the PEG-modified electrode with a molecular weight of 3400 Da has the strongest ECL strength, as shown in Figure 3C. This result confirms that the thickness of the PEG layer plays an important role in the regulation of ECL emissions.

Meanwhile, the density of the PEG chain at the electrode affects the collapsing of the chain and the following collision of Ru(bpy)^2+^ towards the electrode surface. In particular, a low density of PEG chains introduces a lower amount of the immune complex at the electrode, while a high density of the PEG chain induces a crowded environment that does not permit the collision of Ru(bpy)^2+^ completely. To evaluate this effect, different concentrations of PEG (3400 Da) were modified on the electrode. The ECL measurement exhibits the strongest emission in line with the concentration of PEG at 10 mM, as shown in Figure 3D. Under this condition, it is expected that the distance between individual PEG chains will be optimized to achieve the highest efficiency of Ru(bpy)^2+^ collision at the electrode.

### 3.3. Detection of GADA in Serum

**Figure 4:**
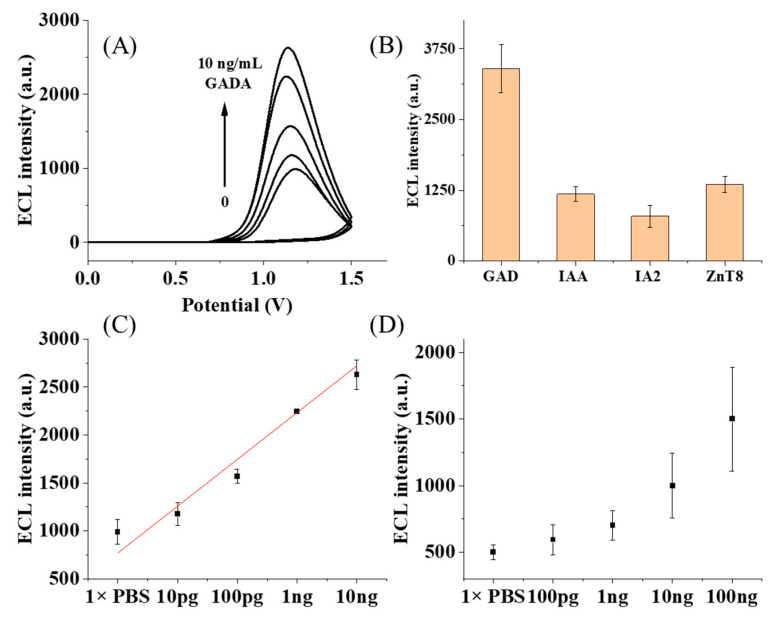
(**A**) ECL responses of the immunosensor toward diverse concentrations of GADA modified with HS-PEG_(3400)_-COOH; (**B**) the selectivity of the immunosensor with various autoantibodies (GADA, IAA Ab, IA2 Ab and ZnT8 Ab); (**C**) the calibration curve of the ECL immunosensor for GADA modified with HS-PEG_(3400)_-COOH; and (**D**) the calibration curve of the ECL immunosensor for GADA modified with TGA at various concentrations.

Under the optimum fabrication conditions, the immunosensors are exposed to different concentrations of GADA, and the ECL intensity from the formed immune complex is recorded. With more GADA, the gradual increase in the ECL intensity is observed in Figure 4A, suggesting the sensitive detection of GADA. To investigate the specificity of this sensor, the interfering antibody, as well as protein tyrosine phosphatase antibody (IA-2A), insulin antibody (IAA), or transporter 8 antibody (ZnT8A), are added into the solution for detection. The ECL intensity of GADA is significantly higher than other antibodies, as shown in Figure 4B, supporting the fact that the ECL immunosensor has good selectivity for GADA. This good feature should also be ascribed to the presence of PEG chains at the electrode, which is reported to minimize non-specific protein adsorption [42]. As a non-toxic polymer with the capability to improve the affinity of materials with water, the PEG layer could produce a microenvironment conducive to protein stabilization and improve biomolecular interactions. With the proper chain length, this brush layer should be sufficient for projecting protein–substrate interactions and the block diffusion of non-specific proteins. Consequently, fabricated immunosensors using PEG chains can improve the selectivity of the immunoassay, which is critical for the following serum test.

Figure 4C demonstrates the relationship between the ECL intensity and the GADA concentration from 10 pg/mL to 10 ng/mL. In controlled experiments without GADA, low ECL signals are used as background signals, and the results show that there is no non-specific adsorption on the electrode with the use of PEG. The near-linear dependence confirms the good response of the sensor to GADA. The detection limit is determined to be 10 pg/mL, which is 100-fold lower than the result (5 IU/mL, equivalent to 1 ng/mL) acquired by the classic enzyme-linked immunosorbent assay (ELISA) at hospitals [43,44]. Moreover, compared with ELISA (100 μL), the ECL immune assay (5 μL) allows a ≥20-fold reduction in the volume of serum. By contrast, TGA, as the rigid chain, is also used to fabricate the immunosensor for the detection of GADA. The lowest concentration that is measured is 100 pg/mL, which is less sensitive to the sensor using the flexible PEG chain. Although the ECL detection using either modification layer could exhibit a sensitive detection of GADA, the use of the flexible PEG chain is preferred because it demonstrates a higher detection signal and a lower detection limit. The collapse of flexible PEG chains brings Ru closer to the surface of the Au electrode, which is more favorable to the electron transfer process, leading to the amplification of the signal from the redox system on the electrode surface.

Next, the established ECL immunosensor was used to perform the test of a clinical serum sample. The results indicate that the concentration of GAD65 Ab in the serum was 11.99 ng/mL, which is consistent with the data detected by the Meso Scale Discovery (MSD) ECL assay method. The RSD of the ECL intensity from four measurements was calculated to be 8.69%. The recovery of GADA was determined to be 92%, respectively. According to these evaluations, the developed ECL immunosensor should be capable of detecting GADA in human serum samples.

## 4. Conclusions

In this work, a new ECL immunosensor was constructed by introducing flexible PEG chains at the electrode for the immobilization of the immune complex, which exhibits high detection sensitivity to GADA. The design offers more freedom in the movement of Ru(bpy)_3_^2+^ at the complex so that it can more easily access the electrode for the initial ECL reaction. Compared with the rigid chain, a higher ECL intensity and sensitivity of detection are obtained from the sensor, with the flexible chain supporting this successful design. The detection limit was almost two orders lower than the value using the classic ELISA kit, which offers power in the early diagnosis of T1DM based on the sensitive detection of GADA. This approach can be extended to the analysis of more target proteins to validate this protocol. Moreover, the fabrication of the immune array to detect multiple autogenous antibodies is being conducted in the laboratory using this protocol to achieve a more accurate evaluation of T1DM patients.

## Data Availability

The original contributions presented in this study are included in the article; further inquiries can be directed to the corresponding authors.
